# Effects of Surface Properties on Gastrocnemius Medialis and Vastus Lateralis Fascicle Mechanics During Maximal Countermovement Jumping

**DOI:** 10.3389/fphys.2020.00917

**Published:** 2020-08-31

**Authors:** Enzo Hollville, Giuseppe Rabita, Gaël Guilhem, Jennyfer Lecompte, Antoine Nordez

**Affiliations:** ^1^French Institute of Sport (INSEP), Laboratory Sport, Expertise and Performance (EA 7370), Paris, France; ^2^NG Lab, Natural Grass, Paris, France; ^3^Human Movement Biomechanics Research Group, Department of Movement Sciences, KU Leuven, Leuven, Belgium; ^4^Arts et Métiers ParisTech, LBM/Institut de Biomécanique Humaine Georges Charpak, Paris, France; ^5^Movement – Interactions – Performance, MIP, EA 4334, Université de Nantes, Nantes, France; ^6^Health and Rehabilitation Research Institute, Faculty of Health and Environmental Sciences, Auckland University of Technology, Auckland, New Zealand

**Keywords:** ultrasound, fascicle, surface stiffness, power amplification, electromyography, jumping

## Abstract

Interactions between human movement and surfaces have previously been studied to understand the influence of surface properties on the mechanics and energetics of jumping. However, little is known about the muscle-tendon unit (MTU) mechanics associated with muscle activity and leg adjustments induced by different surfaces during this movement. This study aimed to examine the effects of three surfaces with different properties (artificial turf, hybrid turf, and athletic track) on the muscle mechanics and muscle excitation of the gastrocnemius medialis (GM) and vastus lateralis (VL) during maximal countermovement jumping (CMJ). Twelve participants performed maximal CMJs on the three sport surfaces. GM and VL muscle fascicles were simultaneously imaged using two ultrafast ultrasound systems (500 Hz). MTUs lengths were determined based on anthropometric models and two-dimensional joint kinematics. Surface electromyography (EMG) was used to record GM and VL muscle activity. Surface mechanical testing revealed systematic differences in surface mechanical properties (*P* = 0.006, η^2^: 0.26–0.32, *large*). Specifically, the highest force reduction and vertical deformation values have been observed on artificial turf (65 ± 2% and 9.0 ± 0.3 mm, respectively), while athletic track exhibited the lowest force reduction and vertical deformation values (28 ± 1% and 2.1 ± 0.1 mm, respectively) and the highest energy restitution (65 ± 1%). We observed no significant difference in CMJ performance between the three surfaces (∼35–36 cm, *P* = 0.66). GM and VL fascicle shortening (*P* = 0.90 and *P* = 0.94, respectively) and shortening velocity (*P* = 0.13 and *P* = 0.65, respectively) were also unaffected by the type of surface. However, when jumping from greater deformable surface, both GM muscle activity (*P* = 0.022, η^2^ = 0.18, *large*) and peak shortening velocity of GM MTU (*P* = 0.042, η^2^ = 0.10, *medium*) increased during the push-off phase. This resulted in a greater peak plantar flexion velocity late in the jump (*P* = 0.027, η^2^ = 0.13, *medium*). Our findings suggest that maximal vertical jumping tasks in humans is not affected by common sport surfaces with different mechanical properties. However, internal regulatory mechanisms exist to compensate for differences in surface properties.

## Introduction

Maximal vertical jumping performance mainly depends on the mechanical power generated by the lower limb muscle-tendon units (MTU) during the push-off phase (Bobbert et al., 1986; Anderson and Pandy, 1993; Kurokawa et al., 2003; Farris et al., 2016; Nikolaidou et al., 2017; Wade et al., 2019). As such, jumping movement has been analyzed in light of muscle and tendon behaviors (Bobbert et al., 1986; Anderson and Pandy, 1993; Kurokawa et al., 2003; Farris et al., 2016; Nikolaidou et al., 2017; Wade et al., 2019) and there is evidence for a decoupling mechanism between muscle fascicles and joint motion thanks to the compliance of the tendinous tissues (Alexander, 1974; Holt, 2019). Specifically, tendinous tissues (connective tissues: extracellular matrix, aponeurosis, tendon) can act like springs by storing elastic strain energy and rapidly releasing it to power body movements (Alexander and Bennet-Clark, 1977; Roberts, 2016). This “catapult-like” mechanism allows the amplification of MTUs’ power outputs far beyond the contractile power capabilities of the muscle (Alexander and Bennet-Clark, 1977; Roberts and Azizi, 2011). These mechanical interactions between muscle and tendon are modulated by the nervous system, and the level and timing at which a muscle is activated directly influences power amplification by the tendinous tissues (Anderson and Pandy, 1993; Bobbert et al., 1996; Wade et al., 2019) as well as the direction of energy flow (e.g., from muscle to tendon to body) (Roberts and Azizi, 2011; Roberts, 2019). During jumping, a proximal-to-distal timing of leg muscle excitation patterns from the hip to the ankle was previously reported (Bobbert and van Ingen Schenau, 1988; Voigt et al., 1995). This sequence is similar to the kinematics sequence of joint extension during jumping (Gregoire et al., 1984; Bobbert and van Ingen Schenau, 1988; Voigt et al., 1995) and allows the appropriate transformation of joint rotations into translation of the center of mass upward through the coordinated action of biarticular and monoarticular muscles during push-off (Gregoire et al., 1984; Van Ingen Schenau, 1989).

During terrestrial locomotion, the surface/substrate is loaded under the body weight and can act like as an additional spring in series affecting movement efficiency (Bosco et al., 1997; Ferris and Farley, 1997; Kerdok et al., 2002), intrinsic stability (Daley and Biewener, 2006), energy dissipation (Hollville et al., 2019), and/or performance (McMahon and Greene, 1979; Arampatzis et al., 2004; Reynaga et al., 2019). However, it seems that in humans, varying common indoor and outdoor sports surfaces do not improve or impair maximal jumping and sprinting performance (Stafilidis and Arampatzis, 2007; Malisoux et al., 2017; Firminger et al., 2019; Hatfield et al., 2019). The main reason is probably due to the low contribution of these standardized sports surfaces to the total mechanical work performed by the human body during maximal motor tasks (Arampatzis et al., 2004; Stafilidis and Arampatzis, 2007). A previous study of sprinting on tracks with different degrees of stiffness reported only a minor surface compression (<1 cm) with no effect on sprint performance and leg mechanics (Stafilidis and Arampatzis, 2007). However, internal regulatory mechanisms may be used to maintain similar movement performance with respect to surface characteristics, or the movement could be compromised (e.g., changes in neural and joint coordination can disrupt elastic energy storage and thus affect the tuning of muscle and tendon mechanics; Sawicki et al., 2015; Reynaga et al., 2019; Roberts, 2019).

Humans adjust the way they move depending on the mechanical behavior of the surface (Arampatzis et al., 2004; Stafilidis and Arampatzis, 2007). Surface mechanical behavior is fixed and determined by surface material properties dependent on surface construction (Stafilidis and Arampatzis, 2007; Firminger et al., 2019). While classical body and joint dynamics analyses could not be sufficient to explore such adjustments, they might be detected from neuromuscular and MTU mechanics measures (Hollville et al., 2019). Indeed, we recently observed that surface absorbing capacity can affect muscle-tendon interactions during landing (Hollville et al., 2019). To our knowledge, no study has yet considered these aspects when studying the relation between external environment like sport surfaces and maximal jumping tasks.

The aim of this study was to evaluate the effects of three common sport surfaces (artificial turf, hybrid turf, and athletic track) with different mechanical properties on the fascicle mechanics and muscle excitation level of the gastrocnemius medialis (GM) and vastus lateralis (VL) muscles during maximal vertical jump. We hypothesized that (i) the influence of surface properties would be marginal for jumping performance, and (ii) surface properties would induce changes to jumping neuromechanics via adjustments in fascicle length changes and/or muscle excitation patterns without altering the proximal-to-distal joint sequence.

## Materials and Methods

### Participants

Sixteen active males initially participated in this study after giving written informed consent. Due to issues identified post-data collection (for details, see section “Data Reduction and Statistical Analysis” below), four participants were excluded and our final sample was composed of 12 active males (age: 24.2 ± 2.0 years; height: 178.5 ± 6.4 cm; body mass: 72.7 ± 7.1 kg). The study was conducted in accordance with the Declaration of Helsinki and approved by the local ethics committee (Ouest IV, agreement no. 16/18).

### Experimental Protocol

**FIGURE 1:**
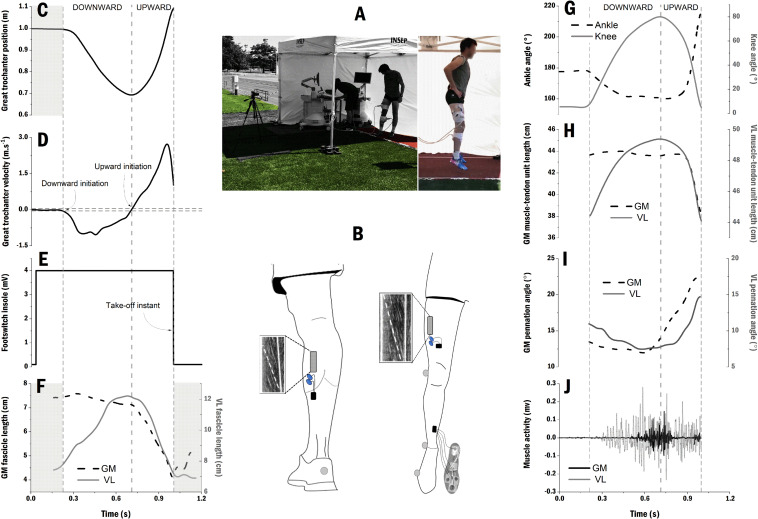
Representative data of one participant and processing overview. **(A)** Experimental setup outdoor under a tent which was moved along the protocol over the three surfaces (i.e., hybrid turf, synthetic turf, and athletic track); **(B)** Positions of the probes and electrodes over the *gastrocnemius medialis* (GM) and *vastus lateralis* (VL) muscle bellies, and the four force resistor sensors which were placed on the insole of the right shoe to detect take-off instant; Great trochanter position **(C)** and velocity **(D)** with downward and upward initiation instants; **(E)** Force resistor sensor pattern with 4 mV corresponding to a contact between the foot and the ground and 0 mv corresponding to flight period; **(F)** GM fascicle length (dash-black line) and VL fascicle length (gray line). The shaded areas correspond to the delimitation of countermovement jump; **(G)** Ankle (dash-black line) and knee (gray line) joint angles; **(H)** GM (dash-black line) and VL (gray line) muscle-tendon units length computed over the countermovement jumps; **(I)** GM (dash-black line) and VL (gray line) pennation angle; **(J)** Raw muscle activity of GM (black) and VL (gray) muscles before processing and normalization procedure (see section “Materials and Methods”).

Data collected for this study is part of a broader protocol comprising other motor tasks analyzed over the same three surfaces (Hollville et al., 2019). We chose to split the data collected in two parts regarding the different hypotheses tested and the amount of information available. We randomized motor tasks as well as the surfaces tested. Experimental protocol was carried out outdoors over three surfaces with different properties (Figure 1A): a third-generation artificial turf (∼40 mm pile height, sand and rubber granules combined as infill, 15 mm shock pad), a hybrid turf (substrate made of cork, sand and micro-synthetic fibers, AirFibr^®^, Natural Grass, Paris, France), and an athletic track (polyurethane). Participants were not specifically familiarized with one surface or another. They warmed up by running 10 min at a self-selected pace and doing dynamic stretching. Then, we placed the ultrasound transducers, EMG electrodes, reflective markers, and insole sensors on the right leg and shoe of each participant. A rapid familiarization (∼20-minute in total) was performed with the realization of maximal countermovement jumps on each surface. We ensured that CMJ performance reached a plateau (i.e., no further increase in jumping height from trial to trial) during the familiarization by providing feedback about the jumping technique (jumping with arms restricted, trunk and legs fully extended during flight time until ground contact) and flight distance (i.e., maximal height reached in the air). In the meantime, ultrasound images, EMG, and insole sensor signals were checked. In total, all participants performed approximately five countermovement jump (CMJ) on each surface before data collection. Afterward, participants performed three maximal vertical CMJ without arm swing at preferred depths over the three surfaces in a random order while data were collected. Between maximal jumps, participants had a passive rest of ∼90 s while they had ∼10 min between surface conditions, corresponding to the time needed to move the entire setup from one surface to another. All participants were familiar with performing maximal jumping tasks on the surfaces used in the present study, which are commonly used in sport practice. We standardized the shoe model so that all participants wore the same pair of molded football cleats (Adidas X16.FG, Herzogenaurach, Germany).

### Surface Testing

Standardized mechanical tests (i.e., vertical impact tests) were performed by an independent surface testing institution (Novarea, Gellainville, France) to characterize surface behavior under specific loading (Hollville et al., 2019). Vertical deformation, force reduction, and energy restitution of each surface were computed from acceleration-time signals (1000 Hz; Advanced Artificial Athlete device, AAA; Deltec Equipment, Duiven, Netherlands). Briefly, force reduction represents the ability of a surface to reduce an impact load (i.e., 20 kg mass dropped from 55 mm onto a 2000 N/mm stiffness spring linked to a 70-mm diameter spherical plate) and was computed according to the following equation:

(1) $$\begin{array}{c} F\,R=\left[1-\left(\frac{F_{m\,a\,x}}{F_{c\,o\,n\,c\,r\,e\,t\,e}}\right)\right]\times 100 \end{array}$$

where *FR* corresponds to force reduction (in %), *F*_*max*_ is the peak force obtained during the impact test, and *F*_*concrete*_ is a theoretical reference force value for a concrete floor (6760 N). *F*_*max*_ was computed using the following equation:

(2) $$\begin{array}{c} F_{m\,a\,x}=m\times g\times G_{m\,a\,x}+m\times g \end{array}$$

where *G*_*max*_ is the vertical peak acceleration during impact (g), *m* is the mass (i.e., 20 kg) and *g* is the gravitational acceleration (i.e., 9.81 m.s^–2^). Vertical deformation (in mm) is the deformation of the surface under the same applied load in the vertical axis. This was calculated from the time when the spherical plate first contacts the surface until the time of the maximum absolute velocity of the mass using the equation:

(3) $$\begin{array}{c} V\,D=D_{m\,a\,x}-D_{s\,p\,r\,i\,n\,g} \end{array}$$

where *D*_*max*_ is the displacement of the falling mass and *D*_*spring*_ is the displacement of the spring (Colino et al., 2017). Finally, energy restitution (in %) is determined by the energy input minus the amount of energy that has been lost in the surface. The area under the unloading force-deformation curve obtained from acceleration-signals describes the energy return of the surface (Baroud et al., 1999). This value represents the surface ability to return energy after being deformed with 100% corresponding to zero energy loss (i.e., no hysteresis). Three trials per test per surface were averaged. The experiments were performed over 3 weeks with similar forecast conditions. Specifically, the experiments took place in Paris in June in the shade (under tents) with relatively high air temperature (20–34°C). Surface maintenance (substrate decompaction, water spreading, mowing) and surface hardness homogeneity control were performed before and after each protocol, resulting in similar surface conditions between subjects.

### Joint Kinematics

Since it was not possible to use a laboratory motion capture system outdoors, a high-speed video camera (300 frame.s^–1^; Casio Exilim EX-F1, Japan) was used to record the two-dimensional (2D) positions of six reflective markers placed on the right side of the participants at the following locations: the fifth metatarsal, lateral calcaneus, lateral malleolus, lateral femoral epicondyle, great trochanter, and the acromion. The camera axis was perpendicular to the sagittal plane of jumping to prevent from image distortion and inaccurate marker trajectories (Kurokawa et al., 2003; Hickox et al., 2016). Marker trajectories were semi-automatically digitized (Dartfish ProSuite 9.0, Fribourg, Switzerland), low-pass filtered (8 Hz) (Kurokawa et al., 2003) and used to retrieve marker coordinates. Ankle, knee, and hip joint angles and velocity were computed at each time frame during the CMJ, and we identified the onset of joint rotation to investigate the proximal-to-distal joint sequence (Winter, 2009). Four force sensitive resistors (Footswitch FSR sensor, Zerowire, Cometa systems, Milan, Italy; Figures 1B,E) were fixed onto the insole of the right shoe to synchronize jumping take-off with ultrasound data (Figure 1E) and to compute jump height based on flight time (Bosco et al., 1983). The flight time method estimates the flight distance during jumping and does not account for center of mass displacement before take-off. For sake of clarity, we will use the term jump height throughout the manuscript to express the maximum height reached by the center of mass (i.e., flight distance). Simultaneous take-off of both legs was visually and blindly checked by the same investigator. Kinematics data were synchronized at take-off based on the fifth metatarsal *y*-coordinate. The onset of downward motion was identified as when the velocity of the marker attached over the great trochanter was inferior to −0.05 m.s^–1^ (Figure 1D). The onset of push-off phase was identified as when the velocity of the same marker exceeded 0.05 m.s^–1^ (Figure 1D; Farris et al., 2016).

### Muscle-Tendon Unit Mechanics

GM and VL muscle fascicles were simultaneously imaged using two ultrafast ultrasound systems (Aixplorer, Supersonic Imagine, Aix-en-Provence, France) synchronized with a common trigger to a digital converter (DT 9804, Data Translation, Marlboro, MA, United States). Two linear probes (4–15 MHz, SuperLinear 15–4, 55 mm field of view, Vermon, Tours, France) were first placed transversally over GM and VL muscle bellies to identify muscle areas; then the probe was gradually rotated longitudinally so that the probe was aligned with fascicle orientation (Figure 1B), then fastened using custom-made supports, elastic bandages, and tape. Participants were asked to perform low-intensity knee flexions, extensions, plantar flexions, and dorsiflexions in order to ensure that the observable part of the fascicles was clearly visible and that the probe was oriented according to the fascicle’s line of action over the entire movement. The acquisition was performed with the research mode with the following parameters: 500 Hz sample frequency, images acquired over a 2 seconds period of time, gain 30–55 dB, 8–10 MHz of ultrasound frequency. Muscle fascicles and aponeuroses length were tracked on B-mode images using a semi-automatic tracking algorithm previously validated (Cronin et al., 2011; Gillett et al., 2013; Farris and Lichtwark, 2016). For each participant, the same fascicle was identified on the initial image of all trials in order to improve reliability of fascicle length measurements between trials. Fascicle length extrapolations are inherent to fascicle dynamics tracking with single short probe field of view (Kurokawa et al., 2003; Brennan et al., 2017). Trigonometry equations were used when necessary to extrapolate GM and VL fascicle lengths (Kurokawa et al., 2003), which were then reported in both absolute (cm) and relative values (i.e., divided by fascicle length measured during a static trial where participants were standing up). Pennation angle was defined as the angle formed at the intersection between fascicle and deep aponeurosis. For both muscles, the lowest and highest pennation angle was identified and we calculated changes in pennation angle as the difference between the two values. In addition, only for GM muscle, we reported an average pennation angle between 80 and 85% of the CMJ. This latter value corresponds to the pennation angle at the time where the GM tendinous tissue length reached its maximum and GM muscle activity is declining (i.e., end of tendinous tissues energy storage – start of energy release; Kurokawa et al., 2003). MTUs’ lengths were computed at each time point based on joint angles and anthropometric models (Grieve et al., 1978; Visser et al., 1990). MTU and fascicle velocity were derived from their measured lengths. Peak MTU shortening velocity and peak and average muscle fascicle shortening velocity were computed. Furthermore, peak MTU shortening velocity was divided by peak muscle fascicle shortening velocity to obtain MTU gearing. This ratio represents the amplification of MTU velocity owing to tendinous tissues compliance and fascicle rotation (or the product of the belly gearing and the tendon gearing; Wakeling et al., 2011).

### Surface Electromyography

Surface electromyography (EMG) was recorded to measure GM and VL muscle activity using a wireless system (ZeroWire, Aurion, Italy), which was synchronized with the force resistive sensors and the two ultrasounds via an external trigger. Bipolar electrodes were placed longitudinally with respect to the fascicle’s alignment and the ultrasound position (Figure 1B). Raw EMG signals were pre-amplified (input impedance; 20 MM, common mode rejection ratio: 90 dB; signal-to-noise ratio: >50 dB; gain: 1000), digitized at 2000 Hz, and then transmitted wirelessly to a remote unit. Raw EMG data were processed with a custom Matlab script (The MathWorks, Natick, MA, United States). The DC offset was removed from raw signals, then bandpass filtered (10–450 Hz), rectified, and averaged with a rolling root mean squared calculation over consecutive windows of 50 ms. To estimate muscle activity during the countermovement jump, EMG RMS data were averaged in two phases: during the last 100 ms of the countermovement (i.e., downward phase), and during the push-off phase. EMG RMS data were normalized to the averaged EMG RMS values obtained on the stiff athletic track surface in both phases (Moritz et al., 2004).

### Data Reduction and Statistical Analysis

Test-retest repeatability of the maximal CMJ task between trials (data pooled between surface conditions; CV: 3.4%; SEM: 1.84 cm; ICC: 0.977) and for each surface condition (artificial turf, CV: 3.6%; SEM: 1.68 cm; ICC: 0.970; hybrid turf, CV: 4.2%; SEM: 1.92 cm; ICC: 0.980; athletic track, CV: 2.4%; SEM: 1.91 cm; ICC: 0.980) demonstrated good to very good repeatability within-subject on all surfaces. The trial resulting in the highest jump for each surface was used for statistical comparisons. As previously mentioned, due to issues with ultrasound images, four participants among the sixteen initial participants were excluded, resulting in *N* = 12 for fascicle-related data and joint kinematics. These issues were mainly large extrapolation of the fascicle, fascicle/aponeuroses curvature, or out-of-plane images. In addition, due to the challenging aspect of fixing a transducer and EMG electrodes near the same location over the muscle belly, we have prioritized placement of the transducer at the expense of an optimal placement of the electrodes. After EMG signal frequency domain analysis (Fast-Fourier Transform), filtering, and careful visual inspection of the signals, EMG data of two and four participants were faulty for GM and VL, respectively, and were therefore excluded, resulting in *N* = 10 for GM muscle activity and *N* = 8 for VL muscle activity.

All variables were analyzed from the onset of downward motion initiation until the point of take-off (Figure 1). Statistical analysis was performed using Origin software (Origin Pro 2018, OriginLab Corporation, Northampton, MA, United States). The effects of surface properties on MTU mechanics (i.e., GM and VL MTUs and fascicle length changes, pennation angle, average and peak shortening velocity and, MTU gearing) and joint kinematics (i.e., range of motion, angular velocity, timing of joint extension) were tested via one-way repeated-measures ANOVA. Kruskal-Wallis non-parametric tests with multiple comparisons were completed to determine the effect of type of surface on muscle excitation (EMG RMS amplitude). Non-parametric tests were also performed on surface mechanical parameters (force reduction, vertical deformation, energy restitution). For ANOVAs, a Greenhouse-Geisser correction was performed when sphericity was violated. Bonferroni *post hoc* tests were used when the results were statistically significant (i.e., *P* ≤ 0.05). All grouped data are presented as means ± standard deviations (SD) and confidence intervals (CI 95%). We calculated partial eta-squared (η^2^) as a measure of the effect size for significant results with η^2^ < 0.06 considered as a small effect size, 0.06 < η^2^ < 0.14 a medium effect, and η^2^ > 0.14 a large effect.

## Results

Mechanical testing revealed systematic differences in the resulting mechanical characteristics between the three surfaces (all *P* values = 0.006, η^2^: 0.26–0.32, *large*; Table 1). The highest force reduction and vertical deformation values have been observed on artificial turf (65 ± 2% [CI: 62.7; 67.3], 9.0 ± 0.3 mm [CI: 8.7; 9.3]), while athletic track exhibited the lowest force reduction and vertical deformation values (28 ± 1% [CI: 25.7; 30.3], 2.1 ± 0.1 mm [CI: 2.0; 2.2]) and the highest energy restitution (65 ± 1% [CI: 63.9; 66.1]).

**TABLE 1:** Surface mechanical properties and kinematics data during maximal countermovement jumping over artificial turf, hybrid turf and athletic track.

Parameters	Artificial turf	Hybrid turf	Athletic track	Statistics	
	Mean ± SD [CI 95%]	Mean ± SD [CI 95%]	Mean ± SD [CI 95%]	*P*-values	η*^2^*
***Surface mechanical behavior***					
Force reduction (%)	65 ± 2[62.7;67.3]*#	55 ± 2[52.7;57.3]*	28 ± 1[25.7;30.3]	**0.006**	*0.26*
Vertical deformation (cm)	9.0 ± 0.3[8.7;9.3]*#	6.3 ± 0.5[5.7;6.9]*	2.1 ± 0.1[2.0;2.2]	**0.006**	*0.32*
Energy restitution (%)	38 ± 2[35.7;40.3]*#	29 ± 2[26.7;31.3]*	65 ± 1[63.9;66.1]	**0.006**	*0.32*
***Kinematics***					
Jump height (cm)	35.7 ± 5.8[32.0;39.4]	35.3 ± 6.6[31.1;39.5]	35.9 ± 6.6[31.7;40.1]	0.66	
Downward phase duration (s)	420 ± 53[383;450]	418 ± 49[384;446]	432 ± 48[395;471]	0.52	
Push-off phase duration (s)	251 ± 37[227;274]	246 ± 37[222;269]	259 ± 45[230;288]	0.23	
Ankle dorsiflexion (downward, in °)	23 ± 4[20;25]	23 ± 3[21;24]	22 ± 4[19;25]	0.80	
Knee flexion (downward, in °)	72 ± 11[65;79]	68 ± 12[61;76]	71 ± 13[63;80]	0.25	
Hip flexion (downward, in °)	87 ± 14[78;96]	87 ± 14[78;96]	87 ± 13[79;96]	0.97	
Ankle plantar flexion (push-off, in °)	56 ± 6[52;60]	55 ± 5[52;59]	56 ± 7[51;60]	0.88	
Knee extension (push-off, in °)	74 ± 10[68;80]	73 ± 10[67;80]	75 ± 12[67;83]	0.61	
Hip extension (push-off, in °)	83 ± 11[76;90]	84 ± 10[78;90]	84 ± 10[77;90]	0.87	
Ankle peak extension velocity (in °/s)	641 ± 51[608;673]	646 ± 53[613;679]*	603 ± 53[569;636]	**0.027**	*0.13*
Knee peak extension velocity (in °/s)	656 ± 70[612;701]	677 ± 73[630;723]	658 ± 69[615;702]	0.31	
Hip peak extension velocity (in °/s)	526 ± 49[495;557]	528 ± 46[499;557]	524 ± 44[496;551]	0.87	

***Joint extension sequence***					

**Hip extension corresponds to 0% of the push-off phase**					
Knee (% of the push-off phase)	16 ± 5[13;19]	13 ± 3[11;15]	18 ± 5[15;21]	0.47	
Ankle (% of the push-off phase)	33 ± 9[27;39]	35 ± 7[31;39]	30 ± 10[24;36]	0.33	

*Significantly different from the athletic track. ^#^Significantly different from the hybrid turf. Values are presented as mean ± SD [confidence interval CI 95%]. Statistical significance was set at *P* < 0.05. Partial eta-squared η^2^ is a measure of the effect size with η^2^ < 0.06 considered as a small effect size, 0.06 < η^2^ < 0.14 a medium effect, and η^2^ > 0.14 a large effect.

The CMJ performance was not significantly different between surfaces (*P* = 0.66) with maximal jump height ranging between 0.35 and 0.36 m (Table 1). Similar jump height was also associated with similar downward (*P* = 0.52) and push-off phase durations (*P* = 0.23). We observed a proximal-to-distal joint sequence with hip extension initiating the push-off, followed by knee extension (at ∼15% of the push-off phase) and ankle plantar flexion (at ∼33%; Table 1). This joint sequence was not influenced by the type of surface, as revealed by a constant timing of joint extension across joints (*P* values ranged from 0.33 to 0.69; Table 1). Joint range of flexion during the downward phase (ankle: *P* = 0.80; knee: *P* = 0.25; hip: *P* = 0.97) as well as joint range of extension during the push-off phase (ankle: *P* = 0.88; knee: *P* = 0.61; hip: *P* = 0.87) were not affected by the type of surface. We also observed a significant difference in ankle peak angular velocity during the push-off phase (*P* = 0.027, η^2^ = 0.13, *medium*). Specifically, the hybrid turf exhibited a significant higher ankle plantar flexion velocity compared to the athletic track (7 ± 9%; *P* = 0.041) while we observed a trend for an increase on the artificial turf compared to the athletic track (6 ± 8%; *P* = 0.085) (Table 1). No difference between surfaces was observed for peak angular velocity at the knee and hip joints (*P* = 0.31 and *P* = 0.87, respectively).

**FIGURE 2:**
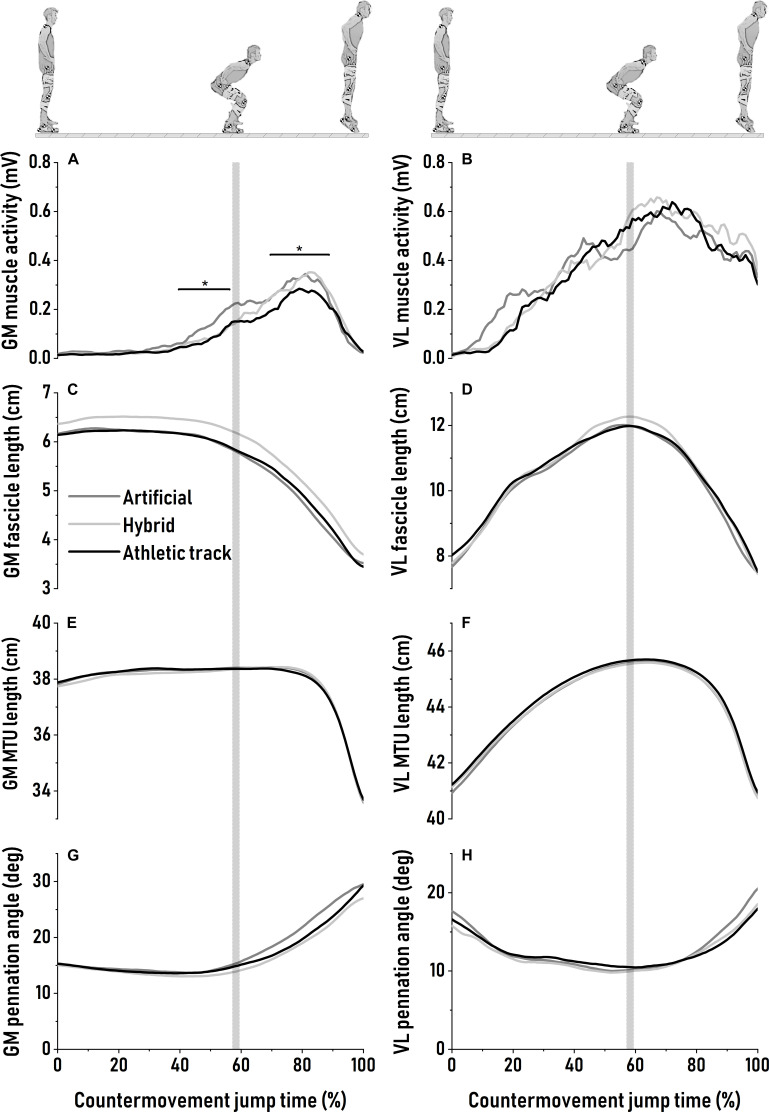
Averaged patterns of *gastrocnemius medialis* muscle activity **(A)**, fascicle **(C)**, muscle-tendon unit **(E)** length changes and pennation angle **(G)** during maximal countermovement jumps on artificial turf (dark gray), hybrid turf (light gray) and athletic track (black). Averaged patterns of *vastus lateralis* muscle activity **(B)**, fascicle **(D)**, muscle-tendon unit **(F)** length changes and pennation angle **(H)** during the same movement over the same surfaces. Standard deviations are omitted for clarity. The vertical shaded area represents the average turning point between the end of the downward phase and the beginning of the push-off phase. Significant bars (*) indicate a significant difference from athletic track.

The patterns of GM and VL MTU and fascicle length are depicted in Figure 2. During the downward phase, GM fascicles operated near-isometrically before starting to actively shorten (on average: −2.5 ± 0.6 cm; *P* = 0.90) at the end of the downward motion (Figure 2C). GM pennation angle was the lowest at ∼40 to 45% at the onset of GM EMG activity rise and when fascicle begins to shorten. No difference in minimum value of pennation angle was found between surfaces (*P* = 0.74, Figure 2G and Table 2). Similarly, for VL, pennation angle reached a minimum value around the transition between countermovement and push-off phase with no surface effect (*P* = 0.78, Figure 2H and Table 2). GM muscle activity was significantly different between surfaces during the last 100 ms of the downward motion, with higher EMG RMS amplitude on the artificial turf than on the athletic track (*P* = 0.047, η^2^ = 0.16, *large*; Figure 2A). During the downward phase, GM MTU length remained almost constant (Figure 2E) while VL MTU lengthened without surface effect (*P* = 0.47; Figure 2F and Table 2). This lengthening was mainly driven by active fascicle lengthening (Figure 2D) with similar lengthening amplitude (*P* = 0.77) between surfaces (Table 2).

**TABLE 2:** Muscle-tendon related-variables (*n* = 12) and muscle excitation amplitude (*n* = 10 in gastrocnemius medialis and 8 in vastus lateralis) during maximal countermovement jumping over three surfaces (artificial turf, hybrid turf and athletic track).

Parameters	Artificial turf	Hybrid turf	Athletic track	Statistics	
	Mean ± SD [CI 95%]	Mean ± SD [CI 95%]	Mean ± SD [CI 95%]	*P*-values	η*^2^*
***Gastrocnemius medialis***					

**Muscle-tendon unit behavior**					
Shortening amplitude (cm)	−4.99 ± 0.88[−5.55;−4.43]	−4.88 ± 0.67[−5.31;−4.45]	−4.87 ± 0.74[−5.43;−4.40]	0.68	
Peak shortening velocity (cm.s^–1^)	−64.1 ± 8.4[−69.4;−58.7]*	−63.9 ± 6.1[−67.7;−60.0]*	−59.0 ± 7.1[−63.5;−54.4]	**0.042**	*0.10*
**Fascicle behavior**					
Shortening amplitude (cm)	−2.51 ± 0.58[−2.88;−2.14]	−2.55 ± 0.64[2.95;−2.14]	−2.49 ± 0.68[−2.92;−2.06]	0.90	
Shortening amplitude (L/Lstanding)	−0.40 ± 0.11[−0.47;−0.33]	−0.39 ± 0.11[−0.46;−0.33]	−0.39 ± 0.09[−0.45;−0.34]	0.84	
Peak shortening velocity (cm.s^–1^)	−16.1 ± 5.0[−19.3;−12.9]	−18.9 ± 7.8[−23.8;−13.9]	−18.2 ± 6.9[−22.5;−13.8]	0.13	
Mean shortening velocity (cm.s^–1^)	−8.3 ± 2.3[−9.8;−6.9]	−9.4 ± 2.5[−11.0;−7.8]	−8.9 ± 3.0[−10.8;−7.1]	0.16	
Muscle-tendon unit gearing	4.4 ± 1.5[3.4;5.3]	3.9 ± 1.6[2.9;4.9]	3.7 ± 1.5[2.8;4.6]	0.20	
Pennation angle (lowest value, deg)	13.3 ± 1.8[11.8;14.7]	12.8 ± 2.2[11.4;14.3]	13.2 ± 3.0[11.8;14.7]	0.74	
Pennation angle (highest value, deg)	29.8 ± 6.0[26.7;33.0]	27.3 ± 5.2[24.1;30.3]	29.5 ± 4.5[26.3;32.7]	0.17	
Changes in pennation angle (deg)	16.6 ± 6.0[13.2;19.9]	14.4 ± 5.5[11.1;17.8]	16.3 ± 4.6[12.9;19.6]	0.09	
Pennation angle at 80–85% CMJ	22.5 ± 4.4[20.7;24.4]	19.5 ± 2.0[17.6;21.4]	20.3 ± 2.8[18.4;22.2]	0.09	

***Vastus lateralis***					

**Muscle-tendon unit behavior**					
Lengthening amplitude (cm)	4.71 ± 0.63[4.31;5.10]	4.59 ± 0.70[4.12;5.03]	4.58 ± 0.68[4.15;5.02]	0.47	
Shortening amplitude (cm)	−4.82 ± 0.47[−5.12;−4.52]	−4.85 ± 0.60[−5.23;−4.48]	−4.76 ± 0.46[−5.06;−4.47]	0.62	
Peak shortening velocity (cm.s^–1^)	−52.0 ± 5.6[−55.6;−48.5]	−53.8 ± 6.4[−57.9;−49.7]	−51.6 ± 5.0[−54.8;−48.4]	0.27	
**Fascicle behavior**					
Lengthening amplitude (cm)	4.16 ± 1.28[3.35;4.98]	4.42 ± 1.43[3.50;5.33]	4.08 ± 1.98[2.83;5.34]	0.77	
Lengthening amplitude (L/Lstanding)	0.53 ± 0.16[0.43;0.63]	0.53 ± 0.13[0.45;0.62]	0.47 ± 0.17[0.36;0.58]	0.34	
Shortening amplitude (cm)	−4.47 ± 1.29[−5.29;−3.65]	−4.60 ± 1.29[−5.23;−3.69]	−4.33 ± 1.62[−5.36;−3.30]	0.94	
Shortening amplitude (L/Lstanding)	−0.57 ± 0.17[−0.68;−0.46]	−0.56 ± 0.14[−0.65;−0.47]	−0.51 ± 0.16[−0.61;−0.41]	0.43	
Peak shortening velocity (cm.s^–1^)	−37.0 ± 15.8[−47.6;−26.4]	−33.7 ± 10.6[−40.8;−26.5]	−35.7 ± 17.5[−47.5;−24.0]	0.65	
Mean shortening velocity (cm.s^–1^)	−16.8 ± 5.6[−20.4;−13.3]	−18.3 ± 6.8[−22.6;−14.0]	−15.7 ± 7.1[−20.2;−11.2]	0.39	
Muscle-tendon unit gearing	1.7 ± 0.6[1.3;2.1]	1.8 ± 0.8[1.3;2.3]	1.8 ± 0.9[1.2;2.4]	0.78	
Pennation angle (lowest value, deg)	9.8 ± 2.0[8.3;11.2]	9.4 ± 2.6[8.0;10.9]	10.1 ± 2.9[8.6;11.5]	0.78	
Pennation angle (highest value, deg)	20.8 ± 5.1[17.6;23.9]	19.0 ± 4.9[15.9;22.1]	19.2 ± 5.1[16.0;22.3]	0.15	
Changes in pennation angle (deg)	11.0 ± 4.8[8.2;13.8]	9.6 ± 5.0[6.8;12.4]	9.1 ± 3.8[6.3;11.9]	0.14	
***Myoelectrical activity***					
Gastrocnemius medialis (%; downward phase)	155 ± 59[114;196]*	122 ± 55[84;160]	100 ± 0	**0.047**	*0.16*
Vastus lateralis (%; downward phase)	127 ± 40[96;158]	103 ± 22[86;120]	100 ± 0	0.31	
Gastrocnemius medialis (%; upward phase)	129 ± 34[105;153]*	128 ± 42[99;157]	100 ± 0	**0.022**	*0.18*
Vastus lateralis (%; upward phase)	99 ± 25[80;118]	107 ± 21[91;123]	100 ± 0	0.64	

* Significantly different from athletic track. A negative value of length changes corresponds to shortening. Values are presented as mean ± standard deviation SD [confidence interval CI 95%]. Statistical significance was set at *P* < 0.05. Partial eta-squared η^2^ is a measure of the effect size with η^2^ < 0.06 considered as a small effect size, 0.06 < η^2^ < 0.14 a medium effect, and η^2^ > 0.14 a large effect. Muscle excitation is expressed as a percentage of the average root-mean-square values obtained for both phases (i.e., downward and upward) on the athletic track. Thus, muscle excitation amplitude on athletic track corresponds to 100%.

During the subsequent push-off phase, GM and VL fascicles actively shortened until take-off without being affected by surface properties (*P* = 0.90 and *P* = 0.94, respectively; Figures 2C,D and Table 2). However, we observed a significant higher GM activation during push-off on the artificial turf (*P* = 0.043) compared to the athletic track while there was a trend for a greater activity also on the hybrid turf (*P* = 0.052) (*P* = *0.022*; η^2^ = 0.18, *large*, Table 2 and Figure 2A). On the contrary, no difference was reported in VL muscle excitation between surfaces during the downward phase (*P* = 0.31) and push-off phase (*P* = 0.64). For both muscles, pennation angle peaked prior to take-off and did not differ between surfaces (Figures 2G,H and Table 2; *P* = 0.17 and *P* = 0.015, for GM and VL, respectively). Similarly, the type of surface did not influence the changes in pennation angle (Table 2; *P* = 0.09 and *P* = 0.014, for GM and VL, respectively). Average GM pennation angle between 80 to 85% of the CMJ was not statistically different between surfaces (*P* = 0.09; Table 2 and Figure 2G) despite a trend for greater pennation angle on artificial turf compared to hybrid turf and the athletic track at this specific moment of the task (∼11–15%; η^2^ = 0.19, large). Neither GM and VL peak (*P* = 0.13 and *P* = 0.65, respectively, Table 2) and average (*P* = 0.16 and *P* = 0.39, respectively, Table 2) fascicle shortening velocity were influenced by the type of surface. GM MTU started to shorten between 75 and 80% of the CMJ (Figure 2E) and shortened with the same amplitude between surfaces (*P* = 0.68), but at a different shortening velocity (*P* = 0.042; η^2^ = 0.10, *medium*). Specifically, we observed a greater peak shortening velocity of the GM MTU on artificial turf (*P* = 0.042) and a trend for a greater velocity on the hybrid turf (*P* = 0.053) compared to the athletic track (Table 2). During push-off, VL MTU shortening and peak shortening velocity were similar between surfaces (*P* = 0.62 and *P* = 0.27, respectively, Figure 2F and Table 2). MTU gearing of the GM revealed a ∼4-fold greater MTU velocity than fascicles but was unaffected by surface properties (*P* = 0.20; Table 2). This MTU gearing ratio was much lower for the VL (∼1.8-fold) and remained similar between surfaces (*P* = 0.78; Table 2).

## Discussion

Despite different measurable surface mechanical properties, we observed no difference in maximal vertical jumping performance between surface conditions. We also found no influence of surface properties on muscle fascicle behavior of both GM and VL muscles during maximal countermovement jumping. However, we observed that surface properties altered GM muscle activation amplitude, which was higher on more absorbing and deformable surfaces such as turf. In addition, the shortening velocity of GM MTU achieved during the push-off phase of the jump was higher on artificial turf and hybrid turf than on athletic track. These adjustments ultimately resulted in greater peak ankle plantar flexion velocity on those surfaces compared to the stiff and less deformable athletic track, and may partially explain neuromechanics’ regulation during jumping to offset changes in surface viscoelastic properties (e.g., increase in damping and compliance).

### Surface Effects on Jump Performance and Kinematics

In accordance with recent studies on both indoor and outdoor sport surfaces (Malisoux et al., 2017; Firminger et al., 2019; Hatfield et al., 2019), jump height was not affected by the type of surface during CMJs. A recent study reported no difference between two different natural turf, an artificial turf, and a force plate (Hatfield et al., 2019) during vertical jumping. These results suggest that the differences in surface mechanical properties between common sport surfaces has only a marginal effect on CMJ performance. This is probably because of a low amount of surface deformation and energy exchange between the human body and the surface, resulting in minor additional work done by the surface, as previously observed in sprinting (Stafilidis and Arampatzis, 2007). We found a greater deformation capacity of the artificial turf compared to the hybrid turf and the athletic track. This can come from different combination of viscoelastic properties and express a global mechanical behavior rather than truly emphasize how surface damping and/or compliance increased. Nonetheless, turf surfaces are certainty more compliant and shock-absorbing than the athletic track. In addition, the higher energy restitution and vertical deformation values of the artificial turf compared to the hybrid turf suggests that the former surface is more deformable and elastic while the latter is more viscous (71% energy loss for hybrid turf vs. 62% energy loss for artificial turf). However, these differences did not impact vertical jump performance.

During CMJ, we observed a proximal-to-distal joint sequence with similar order of lower limb joints extension between surfaces. Our results suggest that jumping coordination remains similar between surfaces. This robust pattern of coordination between the main lower limb joints during jumping is also present when CMJ are performed on stiffer, steel-made force plate or soft and highly deformable sand surface (Giatsis et al., 2018). Similar joint range of motion (i.e., flexion and extension) and time to perform the preparatory countermovement and push-off were found between surfaces, which indicate that participants kept the same jumping strategy whatsoever the type of surface. However, we observed an increase in peak ankle plantar flexion velocity (i.e., reached ∼35 ms before toe-off) on the hybrid turf (+7 ± 9%; significant) and artificial turf (+6 ± 8%; non-significant) compared to the athletic track (Table 1). This higher ankle angular velocity could be possible because of smaller resistance at ankle plantar flexion on the more deformable surfaces (Giatsis et al., 2018). Previously, Giatsis et al. (2018) showed that a decreased resistance due to an increase in surface compliance resulted in both a larger ankle range of motion and angular velocity during CMJ on sand compared to a stiff surface. In our study, it is likely that the greater deformation capacity of the turf surfaces and probably the decreased resistance compared to the athletic track account for the higher peak ankle angular velocity late in the jump. However, the similar ankle range of motion observed between the three surfaces tested suggests that ankle joint excursion is more affected when jumping on a range of very soft surfaces.

### Surface Effect on Muscle Activity

We observed an increase in GM EMG amplitude in both phases when jumps were realized on the more deformable surface with large effect sizes (i.e., artificial turf) (Table 2 and Figure 2A). The mean EMG activity of the GM muscle was on average ∼55% higher on the artificial turf compared to the athletic track, indicating a greater muscle excitation during the countermovement phase. During the first half of push-off, the mean EMG activity of the GM was also approximately 29% and 28% higher on artificial turf and hybrid turf, respectively, compared to athletic track. This result suggests that GM muscle may be more activated in response to an increase in surface deformation capacity (e.g., damping and/or compliance) during maximal vertical jump. It is possible that GM activity is high but submaximal during maximal CMJ, and that there is a potential for further EMG increase through recruitment of additional motor units in order to offset surface properties (Bobbert and van Ingen Schenau, 1988; Kurokawa et al., 2003). Thus, an increase in GM activity may further stiffen the muscle and could be seen as a neural strategy to adjust leg mechanics, and specifically increase ankle stiffness, on a range of compliant surfaces (Moritz et al., 2004). However, further research is needed to explain by which exact mechanisms GM EMG activity increased. VL muscle excitation was similar between surfaces during the countermovement and push-off phases (Figure 2B). Considering similar VL fascicle lengthening during the eccentric part of the CMJ, VL inhibition levels may be comparable when jumping on the three surfaces and could be a reason for the similar level of muscle excitation reported in this phase (Aagaard et al., 2000). In addition, while speculative, VL muscle activity may be near its maximal activation level during the push-off phase of the CMJ, as previously suggested by Nikolaidou et al. (2017). These authors showed that VL fascicles first operated toward optimal length for force generation at the beginning of the push-off phase. Then, when the muscle shortens, it develops high force and mechanical work at a high level of activation (Nikolaidou et al., 2017). In our study, VL fascicle length at the beginning of the push-off phase was similar between surfaces and would not have affected the level of VL activation. Therefore, although we did not assess maximal muscle activity in isometric conditions, further increase in VL muscle excitation may not be possible on more compliant surfaces like turf. Interestingly, we observed no difference in muscle activation/deactivation timings with no longer movement time. This means that the time the muscle actively produces force and work was similar between surfaces. If neural and/or joint coordination or timing were affected by surface properties, it could have consequences for energy flow between muscle and tendinous tissue and potentially affect jumping movement (Sawicki et al., 2015; Reynaga et al., 2019). Our hypothesis is that this is not the case in humans when jumping over these three sport surfaces. In comparison, (Wade et al., 2019) recently demonstrated that adding mass during CMJ increased the time to perform the movement, resulting in lower shortening velocity of gastrocnemius lateralis and soleus muscles, and probably increasing their force and work generation without increasing mean or maximal EMG amplitude. In our study, we could speculate that surface properties tend to affect GM muscle activation level rather than altering the timing of muscle activation and/or the time to perform CMJ. This is also in line with our previous study on the same three surfaces, where we observed an increase in EMG amplitude but no difference in timing of muscle excitation (Hollville et al., 2019). A recent study in animals showed a longer timing of muscle excitation on more compliant habitats, which caused a disruption of the energy flow between the environment and the body, and in turn jumping performance (Reynaga et al., 2019). Any potential changes in muscle excitation timing could differently tune muscle-tendon mechanics and jump height (Sawicki et al., 2015). This may also be a reason to explain the similar muscle fascicle behavior observed in our study. One could wonder how these timings of muscle excitation are affected in humans when jumping from very damped or elastic surfaces, and how it could affect muscle-tendon mechanics and jumping coordination through the proximal-to-distal joint sequence (Ferris and Farley, 1997; Arampatzis et al., 2004; Moritz et al., 2004).

### Surface Effect on Muscle Fascicle Mechanics

Gastrocnemius medialis fascicles were decoupled from the MTU behavior (fascicles were quasi-isometric then shortened whereas MTU lengthened then was quasi-isometric then shortened) (Kurokawa et al., 2003) while we observed that VL fascicles’ behavior (lengthening then shortening) was in phase with the MTU during jumping (Nikolaidou et al., 2017; Figures 2C–F). This result highlights a proximo-distal gradient within the limb to power the jump (e.g., differences in muscle function, tendon compliance) (Roberts, 2016). The higher level of GM activation observed during muscle shortening on the artificial turf (Figures 2A,C) likely increased muscle stiffness increasing the stretch of the tendinous tissues, and in turn the amount of energy stored on this surface (Bosco et al., 1981; Anderson and Pandy, 1993; Farris et al., 2016). Subsequently, elastic energy was released by shortening of the tendinous tissues when GM muscle activity started to decrease (Figure 2A). At this moment, MTU started to shorten (Figure 2E) and the ankle was extended. This catapult-like mechanism potentially amplified mechanical power in GM muscle during push-off (Anderson and Pandy, 1993; Kurokawa et al., 2003; Farris et al., 2016) and may partially explain the increase in peak shortening velocity of GM MTU on artificial and hybrid turf compared to athletic track (Table 2). The higher peak ankle plantar flexion velocity on the hybrid and artificial turf compared to the athletic track observed in the present study might account for such increase in ankle power output. However, it is not clear how there could be an increase in tendinous tissues energy storage and release on turf surfaces. An appealing explanation is related to muscle shape changes and the fact that when a pennate muscle shortens, it radially bulges due to its isovolumetric properties. Such muscle bulging probably exerts forces that load connective tissues not only longitudinally, but also transversely (Azizi and Roberts, 2009; Eng et al., 2018). This biaxial loading likely modulates aponeuroses stiffness in longitudinal and transversal plane (Azizi and Roberts, 2009; Eng et al., 2018). While speculative, additional energy could have been stored through transverse strain on the more deformable surfaces. In addition, the high velocity amplification or MTU gearing of the GM measured as the ratio between MTU and peak fascicle velocity (i.e., GM MTU velocity is ∼four times higher than GM fascicle velocity; Table 2) highlights the key role of tendinous tissues and fascicle rotation to maximize mechanical power during jumping (Alexander and Bennet-Clark, 1977; Anderson and Pandy, 1993). Indeed, this reduction in fascicle velocity is mainly governed by the elastic recoil of the compliant tendinous tissues and to the rotation of GM fascicle. This elastic energy release increases in the late phase of the jump when the force decreases, and thereby likely increases muscle belly thickness (Figure 2G; Kurokawa et al., 2003). While initial GM pennation angle was the same between surfaces at the beginning of the push-off phase, fascicle rotation during the push-off phase appeared to be on average ∼11% to 15% greater on artificial turf than on the other surfaces at the time where tendinous tissues began to release elastic energy (Figure 2G and Table 2). This relative larger (on average 2.2° to 3°) fascicle rotation on artificial turf compared to athletic track and hybrid turf, with similar fascicle length between surfaces, likely increased muscle thickness. The changes in fascicle rotation and muscle belly thickness are considered to be determinants of belly gearing (Wakeling et al., 2011). While speculative, the trend for a greater fascicle rotation and likely muscle thickness observed on artificial turf could result in higher belly gearing on this surface. Such mechanisms may contribute to greater MTU velocity, and to a lesser extent MTU gearing. No significant difference in fascicle length changes and velocity suggest similar muscle fascicle operating length and shortening velocity between surfaces. Previously, Kurokawa et al. (2003) estimated that GM sarcomeres operated on favorable portion of the force-length curve (i.e., over the plateau and upper part of the ascending part) during CMJ, “where fascicles could generate more than 75% of the maximal” fascicle force (Kurokawa et al., 2003). Previous findings in vertical jumping suggest that the difference in muscle architecture and function between the monoarticular soleus and the biarticular GM and gastrocnemius lateralis, both contributing to the generation of ankle power during the push-off phase of the CMJ, may result in different fascicle behavior and elastic mechanisms (Kurokawa et al., 2003; Farris et al., 2016; Wade et al., 2019). It is also possible that soleus fascicle behavior was affected on the more deformable surfaces and contributed to the greater ankle plantar flexion velocity observed in this study.

Vastus lateralis fascicle actively lengthened during the downward motion to resist to inertial and gravitational forces (Nikolaidou et al., 2017). The countermovement allows the VL to produce more positive work during the subsequent push-off phase thanks to the increasing level and development of muscle force (Anderson and Pandy, 1993; Bobbert and Casius, 2005; Figure 2B). This mechanism is attributed to the pre-stretch potentiation of the VL and the ability of a muscle to produce more force after being actively stretched (Bosco et al., 1981), as well as the active muscle state during the preparatory countermovement (Bobbert and Casius, 2005). VL fascicles’ behavior during jumping is similar to a previous study (Nikolaidou et al., 2017; Figure 2D), with high active lengthening and shortening in fascicle and MTU but without difference in magnitude between surfaces (Figures 2D,F and Table 2). In addition, pennation angle decreased throughout the countermovement phase and increased during the push-off until take-off (Figure 2H). However, we found no influence of surface properties (Table 2). This is reliable to the maximal muscle activity assessed on each surface (see previous paragraph) and consistent with previous observations showing that VL fascicles are crucial contributors to the positive mechanical work generation during push-off (Hubley and Wells, 1983; Nikolaidou et al., 2017). Moreover, a previous study during CMJ (Nikolaidou et al., 2017) demonstrated that mean VL fascicle shortening velocity is likely to be close to the plateau of the power–velocity curve and consequently has favorable average power potential. Our current findings show that mean VL fascicle shortening velocity is similar between surfaces and indicate that the sport surfaces tested did not induce changes in VL fascicle mechanics.

In our study, albeit speculative, for similar GM and VL force-length-velocity potentials and contraction history between surface conditions, the overall greater GM EMG activity observed on turf surfaces during the push-off phase suggest a greater GM contractile force output when jumping on artificial turf and hybrid turf compared to athletic track. This would partly explain the similar performances when jumping from more deformable surfaces with higher energy loss potential, likely primarily affecting distal joints and decreasing jumping efficiency (Stefanyshyn and Nigg, 2000; Reynaga et al., 2019).

### Limitations and Methodological Considerations

The results of our study must be considered in light of the following limitations. First, we did not perform *a priori* sample size calculation, which prevents us from determining the power of our sample size. We thus provided confidence intervals (±95%) and effect sizes to better interpret the power of our analyses. Overall, we observed medium to large effect sizes for EMG activity, MTU shortening velocity, and ankle plantar flexion velocity. In addition, our results are clearly supported by previous findings in the field. However, our interpretations/conclusions would be strengthened with a larger sample size. This is especially true regarding non-significant *post hoc* found for ankle peak plantar flexion velocity on artificial turf compared to the athletic track, GM EMG activity during push-off, and GM MTU peak shortening velocity on hybrid turf compared to the athletic track. But maybe more interestingly, this may also hold for non-significant difference observed in GM peak/mean fascicle shortening velocity between surfaces with a small effect size (−11 to −15% less GM fascicle shortening velocity on artificial turf compared to athletic track and hybrid turf; *P* = 0.13–0.16; η^2^ = 0.03–0.04), and GM pennation angle with a large effect size (∼11 to 15% greater pennation angle on artificial at 80 to 85% of the CMJ; *P* = 0.09; η^2^ = 0.19). One could hypothesize that with a bigger sample size, GM fascicle shortening velocity may be slower and GM pennation angle greater during the push-off phase on artificial turf compared to the two other surfaces. Thus, a slower fascicle shortening velocity along with the similar fascicle length and higher EMG activity found would probably enable increased force production on this surface to compensate for greater surface deformation. This could also suggest that an increase in surface deformation may further affect the interplay between GM fascicle and tendinous tissue. These hypotheses need to be confirmed in further studies. Second, we did not use three-dimensional (3D) motion capture system outdoors because of the non-optimal conditions of light for the use of infrared cameras, as well as the need to move our setup two times (from surface to surface) during an entire experiment. However, previous studies reported good agreement between 2D and 3D methods for lower body kinematics and kinetics during jumping (Hickox et al., 2016). Since the CMJ task is mainly restrained to the sagittal plane, we assume that 2D high speed video is appropriate to appraise the influence of different surfaces on 2D joint kinematics (Hickox et al., 2016). Third, we used a simple foot model to capture ankle kinematics, which is then used to compute MTU length. This simple model considers the foot as single rigid-body segment and could have influenced ankle joint and velocity data (Zelik and Honert, 2018). In addition, these estimations failed to account for variability between subjects, and recent studies highlighted their limits of use to estimate tendon work (Zelik and Franz, 2017; Matijevich et al., 2018). Indeed, given the three-dimensional nature of the muscle contraction, the use of 2D ultrasound and anthropometric models to appraise a 3D phenomenon is not without limitations (Cronin and Lichtwark, 2013; Roberts et al., 2019). For example, when a muscle is bulging under contraction, the present simple 2D models may not fully capture transverse strain of the aponeurosis, which partly accounts for longitudinal tendinous tissue length misestimations (Matijevich et al., 2018; Roberts et al., 2019). In this study, we did not estimate tendinous tissue lengths and rather focused on direct estimations of fascicle length to interpret GM and VL tendon function. Additionally, these methods only hold true when the muscle fascicle acts in the same 2D plane as the ultrasound image, thus possibly resulting in underestimation of muscle fascicle length changes when there is out-of-plane muscle motion (which we cannot fully rule out). Recent studies using freehand 3D ultrasound allowed to better understand such dynamic shape changes in skeletal muscle and tendinous tissues (Farris et al., 2013; Raiteri et al., 2018). However, it remains impossible to apply these methods in fast dynamic motor tasks such as jumping. Fourth, we used a fascicle-tracking algorithm previously validated for GM and soleus muscles (Cronin et al., 2011; Gillett et al., 2013; Farris and Lichtwark, 2016), but not VL, for which the validation remains to be done. Due to the small field of view used by the probes to estimate fascicle kinematics, extrapolation of the visible part of the fascicle was done according to aponeuroses motion (Kurokawa et al., 2003). Especially for VL muscle, fascicle length was systematically extrapolated (Brennan et al., 2017). In a pilot analysis (*N* = 1), we found that when using a single short probe, dividing VL fascicle length by a reference VL fascicle length estimated on a quiet standing trial allowed to reduce the percentage of fascicle length extrapolation to 3 to 4% in comparison to VL fascicle length measured with a dual probe arrangement (∼20%; Hollville et al., 2019). This is due to the fact that VL fascicle length value recorded during the static trial was systematically higher using one probe than using two probes which thus reduced the influence of overestimation when using one probe. We found no difference of GM and VL fascicle length changes when using both absolute and relative length values (Table 2). Eventually, the recent study of Brennan et al. (2017) also suggests that the use of a single probe method to estimate VL fascicle length and compare conditions performed in one experimental session is appropriate because of similar differences in muscle contraction dynamics within-participants. Fifth, we restricted the use of arm movement during CMJ in order to mainly examine the contribution of lower-limbs to power the jump. While all participants were familiar with performing maximal vertical jumps, this may appear as a novel task for some of them. However, we ensured that CMJ performance reached a plateau (i.e., no further increase in jumping height from trial to trial) during the familiarization by providing feedback about the jumping technique (jumping with arms restricted, trunk and legs fully extended during flight time until ground contact) and flight distance (i.e., maximal height reached in the air). This was confirmed by the good repeatability of performance whatever the surface during the actual testing (CV < 5%). Sixth, we used the flight-time method to estimate flight distance. This method does not account for the center-of-mass displacement before take-off, and thus underestimates jump height. However, this method remains appropriate to estimate flight distance (Wade et al., 2020), especially outside laboratory conditions, and we observed low variability within-participants. Recent field-based estimates of jump height appear to be an interesting alternative of the use of the flight time method by adding the calculation of an anatomically scaled heel-lift constant to improve jump height estimation (Wade et al., 2020). Lastly, considering the unequal samples for fascicle related data (*N* = 12) and EMG data (*N* = 10 and 8 for GM and VL, respectively), caution should be made when interpreting these variables together.

## Conclusion

We have provided evidence of slight adjustments in the mechanics of the gastrocnemius medialis muscle-tendon unit and muscle activation during maximal vertical jump on surfaces with different mechanical properties without modifying vastus lateralis behavior, jumping performance, and jump coordination. This suggests that the neuromechanics of the jump, especially at the distal joints level, can be affected by surface properties (i.e., increase in surface deformation capacity) during vertical jumping without altering jump height or coordination. These small alterations are mainly explained by a greater gastrocnemius medialis activity and a greater tendinous tissues and gearing contribution to the muscle-tendon unit shortening velocity and to the minor contribution of surface deformation, along with the similar jump coordination strategy used by the participants (e.g., proximal-to-distal joint sequence/neural coordination). Further investigations could extend this first study to a broader range of surface properties (e.g., Moritz et al., 2004) and movements (e.g., running, hopping, drop-jumping, Ferris and Farley, 1997; Kerdok et al., 2002; Arampatzis et al., 2004) in order to establish more general mechanisms about the relationship between surface mechanical behavior and muscle-tendon mechanics and neural control of movement.

## Ethics Statement

The studies involving human participants were reviewed and approved by agreement no. 16/18 (Ouest IV). The patients/participants provided their written informed consent to participate in this study.

## Author Contributions

All authors contributed to conception and design of the study, edited, revised, and approved the final version of the manuscript. EH performed the experiment, processed the data, and wrote the first draft of the manuscript.

## Conflict of Interest

EH was employed by the company Natural Grass as part of a part-funded industrial Ph.D. student program (CIFRE: Convention Industrielle de Formation par la Recherche) with the French National Agency of Research and Technology (ANRT) in collaboration with the French Institute of Sport (INSEP) and the University of Nantes. The remaining authors declare that the research was conducted in the absence of any commercial or financial relationships that could be construed as a potential conflict of interest.
